# A89 EXPLORING THE ENDOSCOPY SEDATION EXPERIENCES OF INDIVIDUALS WITH PEDIATRIC-ONSET INFLAMMATORY BOWEL DISEASE (IBD) AFTER THE TRANSITION TO ADULT CARE: A PILOT STUDY

**DOI:** 10.1093/jcag/gwac036.089

**Published:** 2023-03-07

**Authors:** N Bollegala, A Ramkissoon, Z Mohmand

**Affiliations:** 1 Women's College Hospital; 2 University of Toronto, Toronto, Canada

## Abstract

**Background:**

Different sedation practices are utilized in pediatric and adult endoscopy settings. While pediatric endoscopies are performed under deep sedation, adult endoscopies are often performed under conscious sedation.

**Purpose:**

This study sought to explore the experiences of patients with pediatric-onset IBD that have undergone transfer to adult care and to identify gaps in endoscopy sedation practices that may inform a larger and more comprehensive evaluation of this topic.

**Method:**

A pilot study was performed at Women's College Hospital involving patients aged 18 to 25 years old with a diagnosis of pediatric-onset IBD that received endoscopies in both the pediatric and adult setting. Patients were recruited between June 1 and October 1, 2022. A 36-item questionnaire was distributed assessing sedation methods used in pediatric and adult settings, anxiety, pain, satisfaction during endoscopy and perceptions of transition of care preparation as it pertained to endoscopy. This study was reviewed by institutional authorities at WCH and was deemed not to require REB approval.

**Result(s):**

Twelve patients participated in the pilot survey study (7 Ulcerative colitis, 5 Crohn’s disease) with a mean age of 22. Of those, 100% were female and 66% did not have any comorbid conditions. The mean age of diagnosis was 12.8 years, with respondents spending an average of 67.3 months in pediatric care prior to transition. In pediatric care, the mean number of endoscopies received was 2.3 with a majority performed under deep sedation (83%). Respondents reported being very satisfied with their sedation experience (58%) and experiencing no pain (67%), however mild to severe anxiety was reported (58%). In adult care, mean endoscopies received was 2.1, with conscious sedation (50%) performed more often than deep (42%) or no sedation (8%). More respondents reported satisfaction with deep sedation (100%) than conscious sedation (67%). During conscious sedation, respondents experienced more pain (50% vs. 20%), moderate discomfort (33% vs. 0%), and were more anxious (83% vs. 60%) than in deep sedation. Factors that contributed to anxiety included fear of endoscopy and insufficient sedation. Of those who received deep sedation in the adult setting, 60% reported that the sedation method utilized impacted their future willingness for endoscopy. Lack of communication regarding sedation method, poor experiences with conscious sedation, and the desire to choose the sedation method appropriate for them were areas for improvement.

**Conclusion(s):**

This pilot study demonstrated that IBD patients transferred to adult care receive varying endoscopy sedation methods and experience differential levels of satisfaction favouring deep sedation. This is an important signal that requires larger study.

**Please acknowledge all funding agencies by checking the applicable boxes below:**

None

**Disclosure of Interest:**

None Declared

